# Urban farms in Miami-Dade county, Florida have favorable environments for vector mosquitoes

**DOI:** 10.1371/journal.pone.0230825

**Published:** 2020-04-06

**Authors:** André B. B. Wilke, Augusto Carvajal, Chalmers Vasquez, William D. Petrie, John C. Beier

**Affiliations:** 1 Department of Public Health Sciences, Miller School of Medicine, University of Miami, Miami, FL, United States of America; 2 Miami-Dade County Mosquito Control Division, Miami, FL, United States of America; Swedish University of Agricultural Sciences, SWEDEN

## Abstract

The creation of urban farms in complex urban built environments may create suitable local conditions for vector mosquitoes. Urban farms have been implicated in the proliferation of mosquitoes in Africa, but there is a dearth in the knowledge of their role in the proliferation of mosquitoes elsewhere. In this study, we surveyed two urban farms in Miami-Dade County, Florida. Our results show that urban farms provide favorable conditions for populations of vector mosquito species by providing a wide range of essential resources such as larval habitats, suitable outdoor resting sites, sugar-feeding centers, and available hosts for blood-feeding. A total of 2,185 specimens comprising 12 species of mosquitoes were collected over 7 weeks. The results varied greatly according to the urban farm. At the Wynwood urban farm, 1,016 specimens were collected but were distributed only between 3 species; while the total number of specimens collected at the Golden Glades urban farm was 1,168 specimens comprising 12 species. The presence of vector mosquitoes in urban farms may represent a new challenge for the development of effective strategies to control populations of vector mosquito species in urban areas.

## Introduction

The intensification in urbanization processes is deeply affecting the ecology and behavior of mosquito species in urban environments [1,2]. Vector species that are adapted to urban environments are gradually increasing their range and abundance in urban areas, and as a result, their contact probabilities with humans [3].

Currently, not only more than 80% of the world population is at risk for vector-borne disease transmission [4,5], but former non-endemic areas are experiencing outbreaks more frequently [6–8]. In 2016, a major Zika virus outbreak with more than 1,000,000 confirmed cases and thousands of fetus malformations caused by the virus took the Americas by storm [9–11]. Two cities in the continental United States reported local transmission of Zika virus: Miami, Miami-Dade County, Florida and Brownsville, Cameron County, Texas [12]. In this context, the community composition and year-round abundance of vector species of mosquitoes make Miami-Dade County, Florida a receptive gateway for arbovirus entry to the United States. Since the Zika virus outbreak in 2016 [13], there have been 646 imported arbovirus infections reported in Miami by the Department of Health (DOH) including dengue, Zika, chikungunya, and West Nile virus. From January to September 2019 alone, the DOH reported 236 travel-related cases of dengue, 7 imported cases of chikungunya, and 15 imported cases of Zika in Miami-Dade County. In this period, 12 locally transmitted cases of dengue have also been reported, which led the health officials to issue a mosquito-borne illness alert for the Miami-Dade area [14].

Biotic homogenization processes driven by urbanization are responsible for decreasing the richness of species and increasing the abundance of the few species adapted to thrive in urban environments in a nonrandom process of biodiversity loss [2,3,15–18]. The presence and abundance of vector mosquitoes that are more adapted to urban environments, such as *Aedes aegypti* and *Culex quinquefasciatus*, is on the rise and, as a consequence, the incidence of vector-borne diseases is increasing globally [19–22].

Miami-Dade County is a major gateway to the United States and is especially at high risk for the introduction of arboviruses due to the high flow of people coming and going from endemic areas. Miami has the proper environmental conditions to support vector mosquito populations: average annual temperature ranges around 25°C and cumulative rainfall around 1573 mm; even in winter (December-March) temperature is mild with median values around 15°C and total rainfall around 50 mm [23].

Consequently, several vector mosquitoes can be abundantly found in Miami, including *Ae*. *aegypti* and *Cx*. *quinquefasciatus*, both widely distributed and abundant year-round [16,18] in many different habitats in the urban environments of Miami. Previous studies showed that *Ae*. *aegypti* and *Cx*. *quinquefasciatus* were successfully exploring construction sites and tire shops deeply inserted in highly urbanized areas of Miami [17,24]. *Aedes aegypti* was also found breeding in high numbers in ornamental bromeliads widely used in landscaping in Miami [18,25]. Controlling vector mosquitoes in these problematic habitats is considered essential by the Miami-Dade Mosquito Control Division in the effort to prevent future arbovirus outbreaks.

In this context, the growing trend to consume organic products produced locally has contributed to the growth of urban agriculture worldwide [26,27]. Urban farms are usually small green areas inserted within one city block, in which crops or animal husbandry activities take place. Urban farms can be kept either by communities or individuals and have the goal of cultivating, processing and distributing locally ground or raised food within the nearby urban areas. The creation of urban farms in the already complex urban built environment may represent a new challenge for the effective control of vector mosquitoes in urban environments.

Urban farms have been implicated in the proliferation of mosquitoes in Africa [28,29], but their role in the United States is still unknown. However, it is not uncommon for vector species to exploit multiple and complex habitats and thrive in urban areas [30,31].

Even though urban farms may provide favorable environments for both native and exotic species playing an important role in species conservation, these areas have a wide range of essential resources for vector mosquitoes such as larval aquatic habitats and available hosts for blood-feeding. Moreover, these areas are no-spraying zones due to bee-keeping activities and pesticide-free crops. Understanding the ecology and behavior of mosquito populations in the increasingly more popular urban farm settings is fundamental for the development of vector surveillance and control in urban areas [27].

We hypothesized that urban farms in Miami-Dade County, Florida have the appropriate conditions to support populations of vector mosquito species. Therefore, the objective of this study was to do a cross-sectional survey of two urban farms located in neighborhoods in Miami-Dade County with distinct levels of urbanization to assess if vector mosquitoes can be found in these areas.

## Materials and methods

### Study design

With approximately 3 million residents Miami-Dade is the most populous county in Florida. Miami is a very complex and dynamic city. It is a culturally diverse and touristic city, receiving millions of tourists every year. Miami also has substantial socioeconomic, urban, and land cover heterogeneity [32].

In this study, we surveyed two urban farms in Miami-Dade County, Florida once a week for 7 consecutive weeks from March 19^th^ to May 2^nd^ 2019 for the presence of vector mosquitoes. Since this study posed less than minimal risk to participants and did not involve endangered or protected species the Institutional Review Board at the University of Miami determined that the study be exempted from institutional review board assessment (IRB Protocol Number: 20161212).

### Study areas

**Fig 1:**
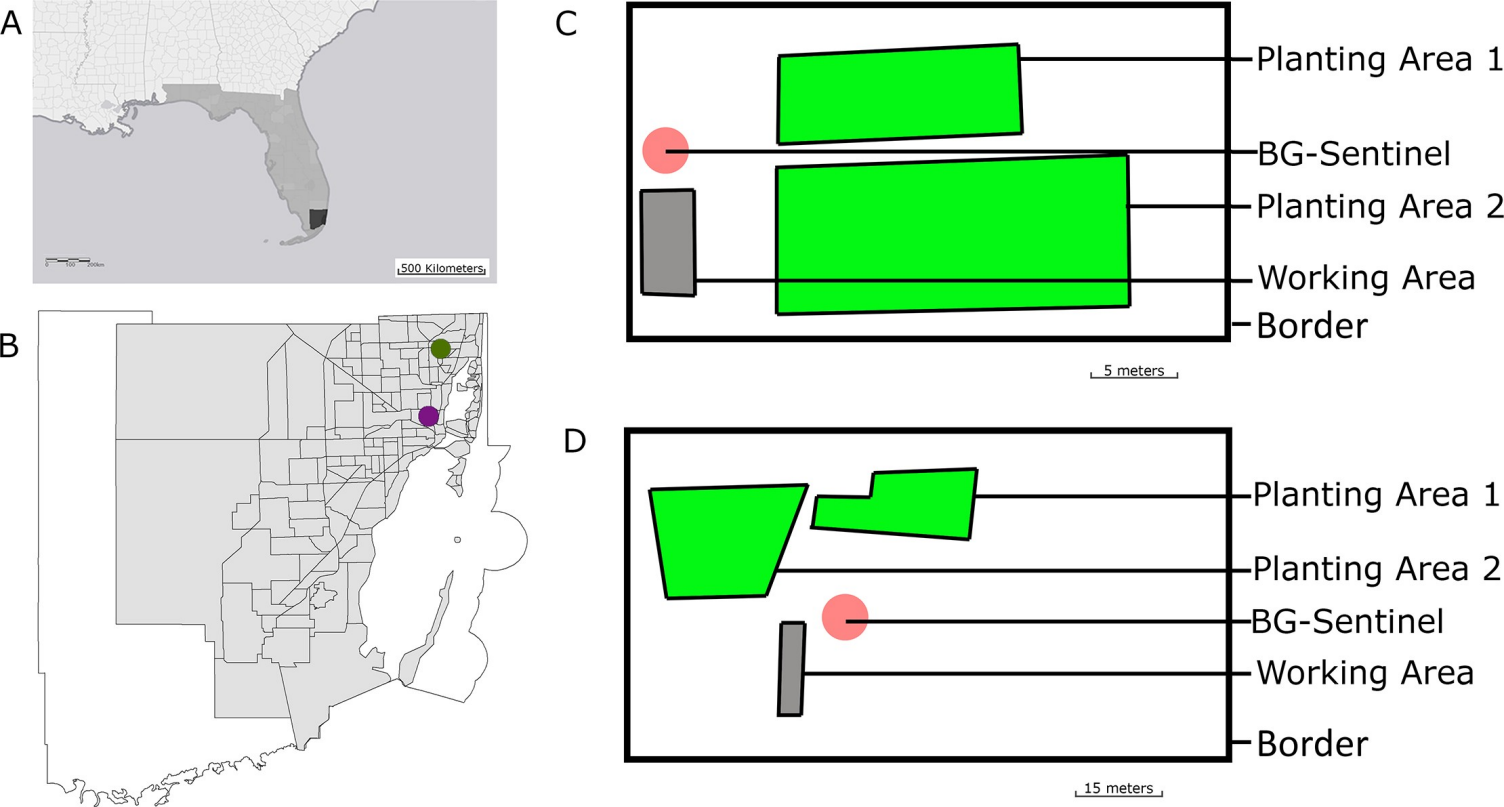
Location of the urban farms in Miami-Dade County, Florida. (A) Southeast United States; (B) Miami-Dade County (Wynwood farm is displayed in purple and Golden Glades farm in green); (C) Wynwood urban farm; and (D) Golden Glades urban farm. Fig 1 was produced using ArcGIS 10.2 (Esri, Redlands, CA) using freely available layers from the Miami-Dade County’s Open Data Hub - https://gis-mdc.opendata.arcgis.com.

Two urban farms within the borders of Miami-Dade County were selected for this study (Fig 1A), one in Wynwood and the other in the Golden Glades (Fig 1B). The urban farm located in Wynwood was selected due to the importance of the area during the Zika virus outbreak in 2016 and due to its high human population density of 12,350 people per square mile and approximately 2 million tourists every year [33]. The Wynwood urban farm has a total surface of 4,675 m^2^ divided into a working area and two planting areas (Fig 1C). The urban farm located in the Golden Glades was considerably larger with 11,900 m^2^ and two different planting areas as well as a working area, and it is located in a suburban area with 6,985 people per square mile (Fig 1D).

A wide range of potential aquatic breeding habitats was available. Both urban farms had two 55 gallons buckets each, one used for water storage and the other as a trash can. The Wynwood farm also had 2 large open planting substrate bags (2.2 cubic feet) which accumulate rainwater, and many pots scattered throughout the farm. The Gold Glades farm had a different scenario with no pots or bags but with a bromeliad patch, a kayak used to harvest rainwater, and a natural pond.

### Adult mosquito and larval sampling techniques

One BG-Sentinel trap (Biogents AG, Regensburg, Germany) was deployed weekly for 24 hours on both urban farms. All traps were baited with CO_2_ using a container filled with 1 Kg of dry ice pellets [34]. On each farm, we sampled mosquitoes in available ground-level vegetation using a pro Improved Prokopack Aspirator (model 1419) with a series of timed 10-minute collections per both working and planting areas. Resting mosquitoes were collected once a week in each farm totaling 30 minutes per week in each urban farm (1 working area and 2 planting areas). Aquatic breeding habitats were surveyed for immature mosquitoes once a week for two hours or until all potential breeding sites were exhausted. Collections of immature mosquitoes were made with entomological dippers and collected mosquitoes were conditioned in 100 ml containers for transport. All collected mosquitoes were transported to the Miami-Dade County Mosquito Control Laboratory and morphologically identified using the taxonomic keys of Darsie and Morris [35]. Adult mosquitoes were promptly identified, larvae were kept at room temperature until L4 and then identified, pupae were allowed to emerge as adults and then identified.

### Mosquito control surveillance network

**Fig 2:**
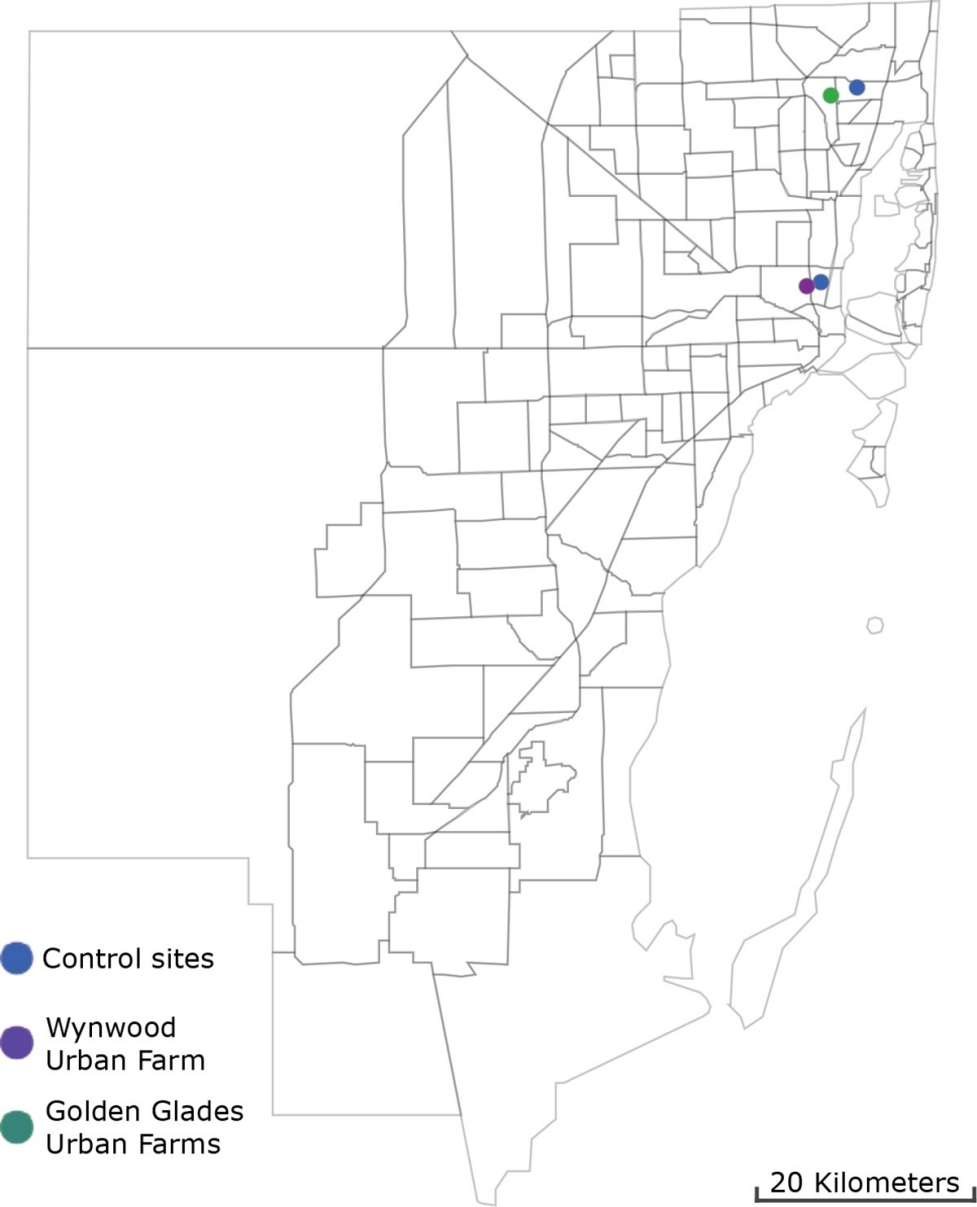
**Map of Miami-Dade County, Florida displaying the location of the Wynwood (purple) and the Golden Glades (green) urban farms in relation to the BG-Sentinel traps from the Miami-Dade County mosquito control surveillance grid (blue).** Fig 2 was produced using ArcGIS 10.2 (Esri, Redlands, CA) using freely available layers from the Miami-Dade County’s Open Data Hub - https://gis-mdc.opendata.arcgis.com.

The Miami-Dade Mosquito Control Network consists of 191 traps (157 BG-Sentinel and 34 CDC traps) deployed weekly for 24 hours since August 2016. All traps are baited with CO_2_, and all collected mosquitoes are transported to the Miami-Dade County Mosquito Control Laboratory and subsequently morphologically identified using taxonomic keys [35]. For this study, we compared the data acquired by two BG-Sentinel traps, one located at 500 meters from the Wynwood urban farm and the other at 800 meters from the Golden Glades Urban farm during the same period of this study (Fig 2). For detailed information on the collection methods refer to Wilke at al (2019) [16].

### Data analysis

After the morphological identification of the mosquitoes to the species level, we carried on with the biodiversity analyses. Biodiversity analyses were done for each urban farm and the control sites individually using the Shannon and Simpson indices [36,37]. Both indices are extensively used to help understand and visualize variations in diversity in an ecological community [38]. The Shannon and Simpson indices are complementary. The Shannon index considers species abundance and communities, less diversity will yield to lower values and vice versa. Simpson (1-D) index estimates species dominance, when close to 1 means the community is mainly dominated by a unique species [39].

To estimate both the sampling sufficiency and the number of species in samples with fewer specimens for all specimens collected in this study we used the species accumulation curve, estimated by individual rarefaction as in Adrain et al [40] using Past software (v.3.16) [41].

Then, the data were analyzed using the cumulative profiles of species log abundance (ln S), Shannon index (H) and log evenness (ln E) (SHE) model. This model computes the ln S, H, and ln E values independently for each sample consecutively to the last sample. Deviations from the straight line are indicative of shifts in species composition and variations in the mosquito assembly [42]. Analyses were carried out with 10,000 randomizations where each randomization is done without replacement using a 95% confidence interval using Past software (v.3.16) [41,43]. To test for differences in the mosquito community between the urban farms and their respective control sites we used the Kruskal-Wallis one-way analysis of variance using Past software (v.3.16).

## Results

A total of 2,185 specimens comprising 12 species of mosquitoes were collected. The five most abundant species were *Cx*. *quinquefasciatus* (1,022 specimens collected, 46.8%), *Cx*. *nigripalpus* (357 specimens, 16.3%), *Ae*. *aegypti* (258 specimens, 11.8%), *Cx*. *coronator* (256 specimens, 11.7%) and *Ae*. *albopictus* (148 specimens, 6.8%) (Tables 1 and 2).

**Table 1:** Mosquitoes collected at the Wynwood urban farm in Miami-Dade County, Florida (USA), from 19/3 to 2/5/2019 in alphabetical order.

Species		Wynwood Urban Farm							
		Week 1	Week 2	Week 3	Week 4	Week 5	Week 6	Week 7	Total
***Aedes aegypti***	BG-Sentinel	20	37	7	1	19	9		93
	Aspirator		2			1			3
	Immature					3			3
***Anopheles quadrimaculatus***	BG-Sentinel								
	Aspirator		1						1
	Immature								
***Culex quinquefasciatus***	BG-Sentinel	77	320	123	61	61	48		690
	Aspirator		18		5	4			27
	Immature				199				199
**Total**		97	378	130	266	88	57		1,016

**Table 2:** Mosquitoes collected at the Golden Glades urban farm in Miami-Dade County, Florida (USA), from 19/3 to 2/5/2019 in alphabetical order. In parenthesis, the number of pupae.

Species		Golden Glades Urban Farm							
		Week 1	Week 2	Week 3	Week 4	Week 5	Week 6	Week 7	Total
***Aedes aegypti***	BG-Sentinel	59	7	7	4	38	27	2	144
	Aspirator				1	4			5
	Immature	2 (7)	1						10
***Aedes albopictus***	BG-Sentinel						5		5
	Aspirator				1				1
	Immature	5	5	102 (3)	12 (13)		2		142
***Aedes taeniorhynchus***	BG-Sentinel			1					1
	Aspirator								
	Immature								
***Aedes tortilis***	BG-Sentinel				20		6		26
	Aspirator								
	Immature								
***Anopheles quadrimaculatus***	BG-Sentinel				1		1		2
	Aspirator								
	Immature								
***Culex coronator***	BG-Sentinel	31	13	26	40	122	17	7	256
	Aspirator								
	Immature								
***Culex erraticus***	BG-Sentinel		8		5	13	17	3	46
	Aspirator								
	Immature								
***Culex nigripalpus***	BG-Sentinel		4	21	156	80	96		357
	Aspirator								
	Immature								
***Culex quinquefasciatus***	BG-Sentinel	23	2	3			14	32	74
	Aspirator	1		7	7	11			26
	Immature						6		6
***Deinocerites cancer***	BG-Sentinel	4						1	5
	Aspirator								
	Immature								
***Wyeomyia mitchelli***	BG-Sentinel		2			15	2		19
	Aspirator				1				1
	Immature			28					28
***Wyeomyia vanduzeei***	BG-Sentinel		1	1		7	6		15
	Aspirator								
	Immature								
**Total**		132	43	199	261	290	199	45	1,169

However, the results varied greatly according to the urban farm monitored. At the Wynwood urban farm, a total of 1,016 specimens were collected and belonged to 3 species: *Cx*. *quinquefasciatus* 916 specimens, *Ae*. *aegypti* 99 specimens and *Anopheles quadrimaculatus* 1 specimen (Table 1). *Cx*. *quinquefasciatus* and *Ae*. *aegypti* were consistently collected in relatively high numbers by the BG-Sentinel trap. Resting *Cx*. *quinquefasciatus* were collected in relatively higher numbers by the manual aspirator, compared to *Ae*. *aegypti*.

A total of 199 immature *Cx*. *quinquefasciatus* and 3 *Ae*. *aegypti* were collected only once from a bucket and a water storage barrel, respectively during the study period (Table 1).

The total number of specimens collected at the Golden Glades urban farm was 1,169 specimens, comprising 12 species (Table 2). The five most abundant species in decreasing order were: *Cx*. *nigripalpus* with 357 specimens, followed by *Cx*. *coronator* with 256 specimens, *Ae*. *aegypti* with 159 specimens, *Ae*. *albopictus* with 148 specimens and *Cx*. *quinquefasciatus* with 106 specimens. Only 4 species were collected from the immature to the adult stages: *Ae*. *aegypti*, *Ae*. *albopictus*, *Cx*. *quinquefasciatus* and *Wyeomyia mitchelli*.

Based on the data obtained from the Miami-Dade Mosquito Control Division surveillance network [16], both the Wynwood and Golden Glades urban farms surveyed in this study not only presented a higher abundance of vector mosquitoes with roughly 5 times more mosquitoes collected using the same sampling effort (one BG-Sentinel trap deployed for 24h and baited with CO_2_) but also higher species richness than their surrounding areas. The total number of mosquitoes collected at the Wynwood control site was 132 whereas 226 mosquitoes were collected at the Golden Glades control site (Tables 3 and 4).

**Table 3:** Adult mosquitoes collected from March 19^th^ to May 2^nd^, 2019 by the Miami-Dade Mosquito control division surveillance BG-Sentinel trap located near the Wynwood urban farm in alphabetical order.

Species	Week 1	Week 2	Week 3	Week 4	Week 5	Week 6	Week 7	Total
***Aedes aegypti***	4	1	4	8	4	1	5	27
***Aedes albopictus***								
***Aedes taeniorhynchus***								
***Aedes tortilis***								
***Anopheles quadrimaculatus***								
***Culex coronator***								
***Culex erraticus***								
***Culex nigripalpus***								
***Culex quinquefasciatus***	29	2	21	3	16	13	21	105
***Deinocerites cancer***								
***Wyeomyia vanduzeei***								
***Wyeomyia mitchelli***								
**Total**	33	3	25	11	20	14	26	132

**Table 4:** Adult mosquitoes collected from March 19^th^ to May 2^nd^, 2019 by the Miami-Dade Mosquito control division surveillance BG-Sentinel trap located near the Golden Glades urban farm in alphabetical order.

Species	Week 1	Week 2	Week 3	Week 4	Week 5	Week 6	Week 7	Total
***Aedes aegypti***	1	6	8	8	14	5	16	58
***Aedes albopictus***								
***Aedes taeniorhynchus***								
***Aedes tortilis***			1					1
***Anopheles quadrimaculatus***								
***Culex coronator***		3			2			5
***Culex erraticus***								
***Culex nigripalpus***								
***Culex quinquefasciatus***	11	13	21	3	62	20	26	156
***Deinocerites cancer***								
***Wyeomyia vanduzeei***		1	1		3	1		6
***Wyeomyia mitchelli***								
***Total***	12	23	31	11	81	26	42	226

**Fig 3:**
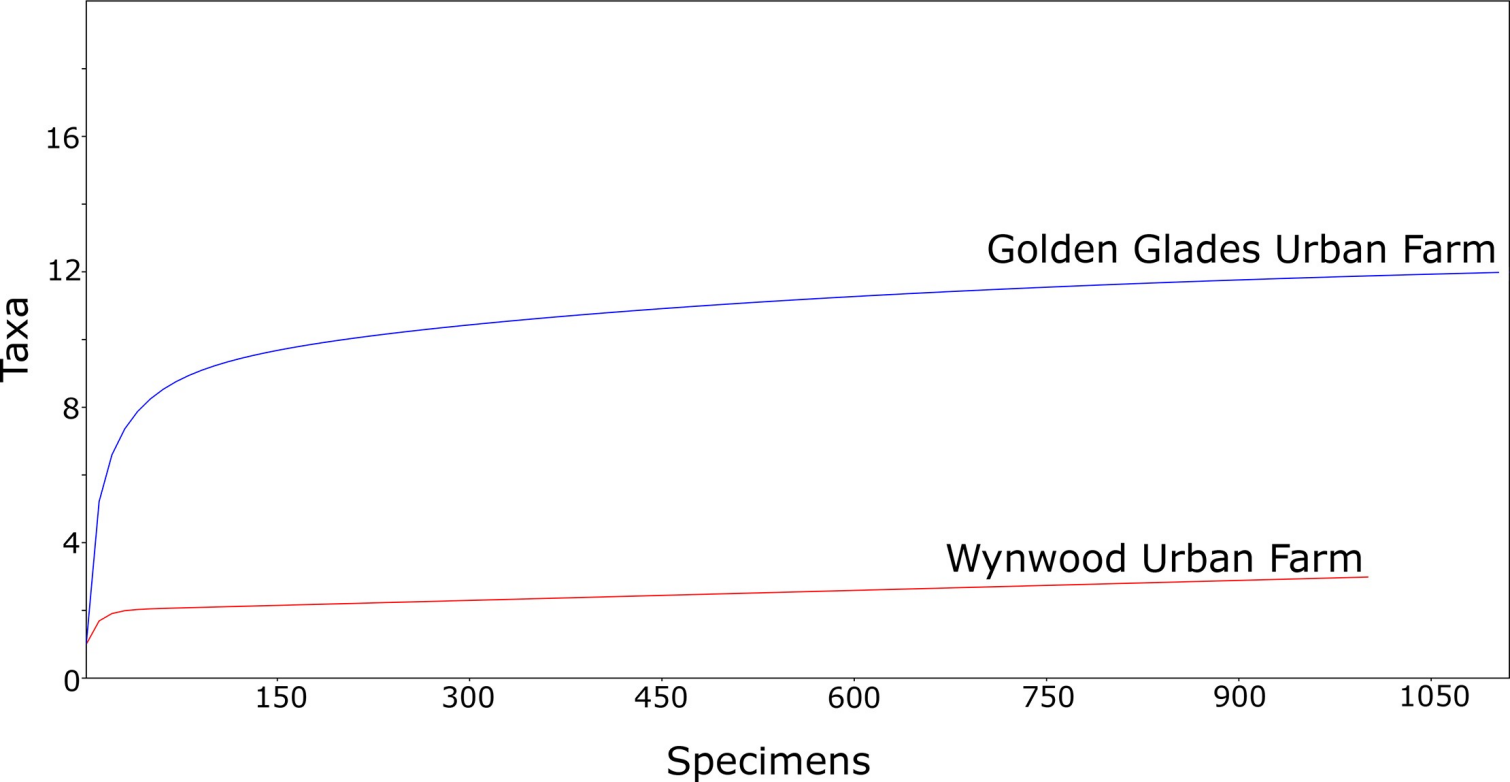
Species accumulation curve for mosquitoes collected at the Wynwood and Golden Glades urban farms in Miami-Dade County, Florida (USA).

The values for Shannon’s diversity index were 0.327 (95% IC: 0.288–0.371) at the Wynwood urban farm and 1.86 (95% IC: 1.812–1.906) at the Golden Glades urban farm. The results for the Simpson (1-D) index were 0.882 (95% IC: 0.791–0.851) at the Wynwood urban farm and 0.195 (95% IC: 0.184–0.208) at the Golden Glades urban farm. Species accumulation curve, estimated by individual rarefaction resulted in a highly asymptotic curve for both the Golden Glades and Wynwood urban farms indicating that sampling sufficiency was achieved for both collection areas (Fig 3).

**Fig 4:**
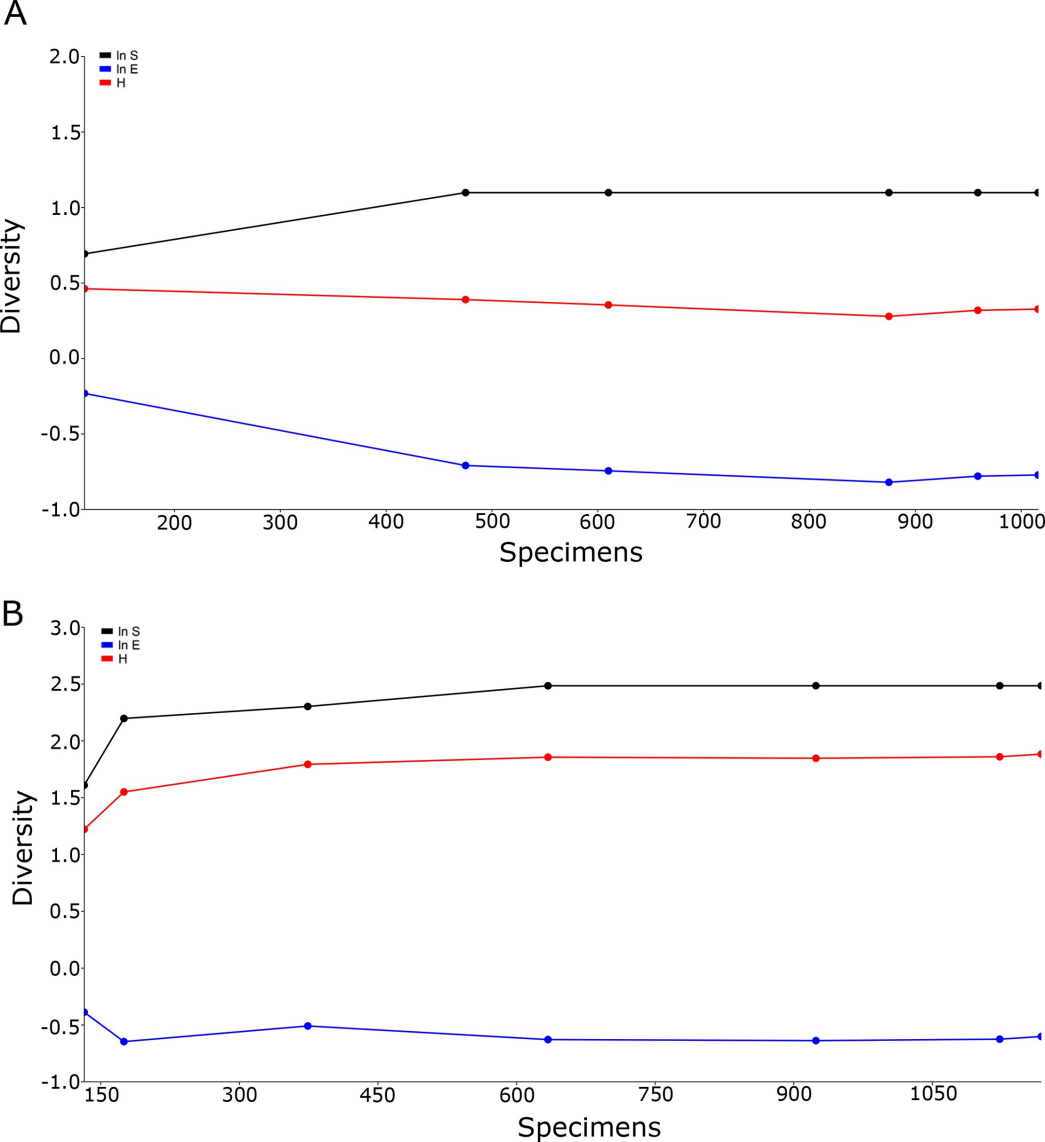
SHE profiles (ln S, H, and ln E) for mosquitoes collected at the Wynwood and Golden Glades urban farms in Miami-Dade County, Florida. (A) Wynwood urban farm; (B) Golden Glades urban farm.

The lack of species richness in the mosquito community found at the Wynwood urban farm resulted in virtually no changes in the direction of the lines in the cumulative SHE analysis, corroborating the lack of variability in species composition, diversity, and evenness (Fig 4A). At the Golden Glades urban farm, after an initial variation in the results of the SHE analysis, no shifts in the direction of the lines of the SHE model were observed, indicating low levels of variability in the mosquito diversity (Fig 4B).

**Fig 5:**
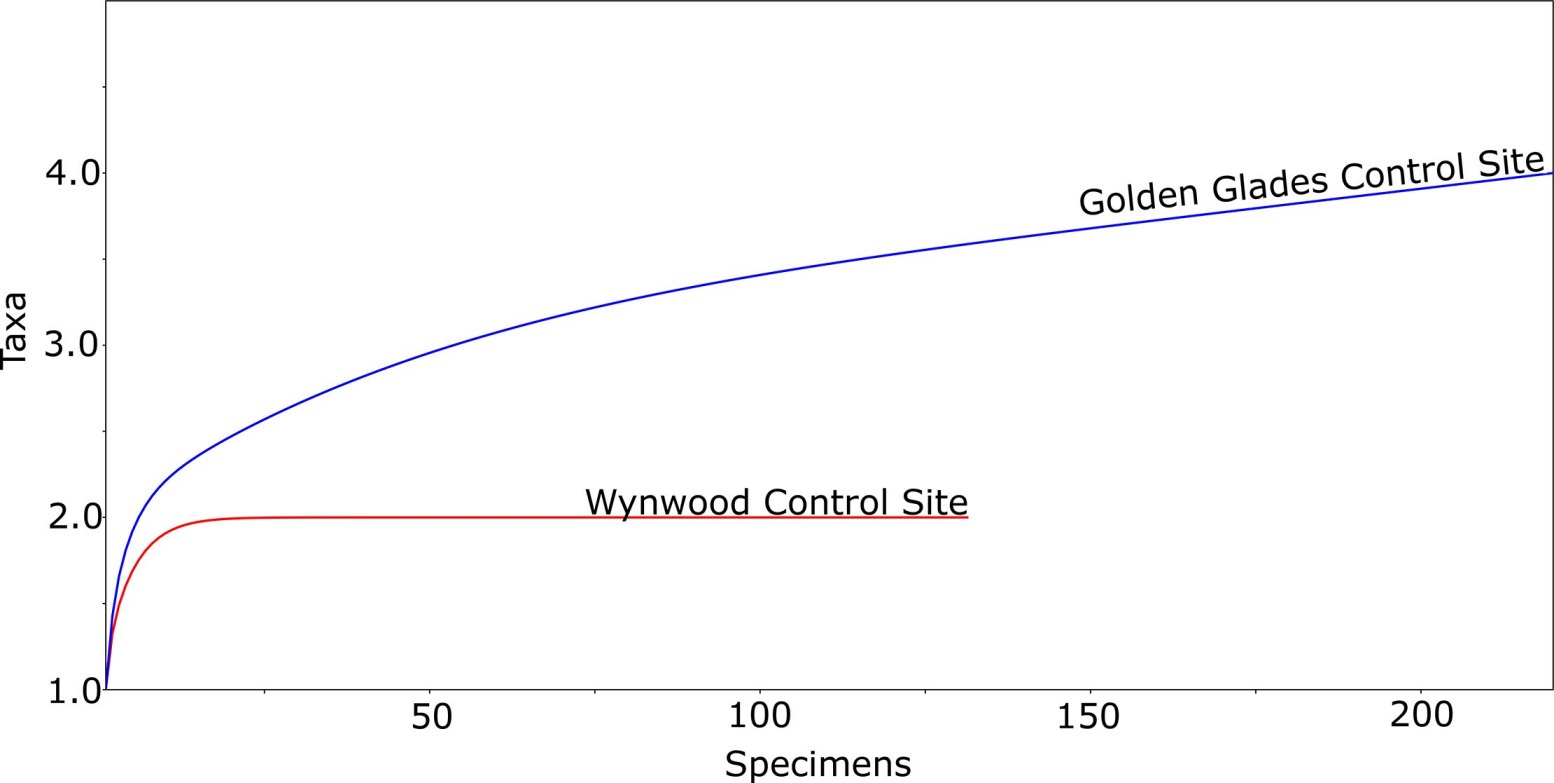
Species accumulation curve for mosquitoes collected at the Wynwood and Golden Glades control sites in Miami-Dade County, Florida (USA).

The values for Shannon’s diversity index were 0.506 (95% IC: 0.398–0.593) at the Wynwood control site and 0.705 (95% IC: 0.621–0.801) at the Golden Glades control site. The results for the Simpson (1-D) index were 0.674 (95% IC: 0.596–0.764) at the Wynwood control site and 0.572 (95% IC: 0.517–0.632) at the Golden Glades control site. Species accumulation curve, estimated by individual rarefaction resulted in a highly asymptotic curve for the Wynwood control site and a moderate asymptotic curve for the Golden Glades control site (Fig 5).

**Fig 6:**
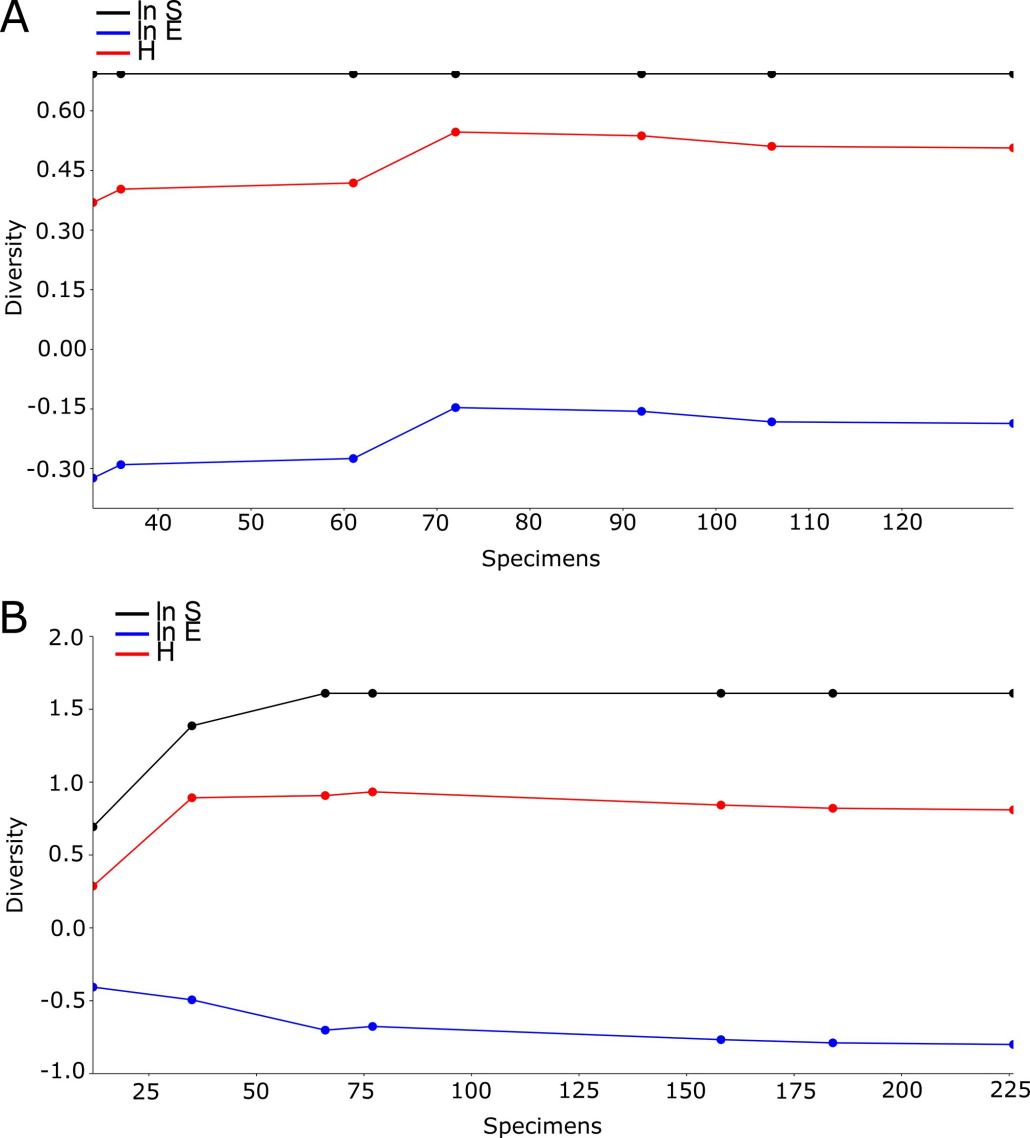
SHE profiles (ln S, H, and ln E) for mosquitoes collected at the Wynwood and Golden Glades control sites in Miami-Dade County, Florida. (A) Wynwood control site; (B) Golden Glades control site.

The cumulative SHE analysis of the Wynwood control site resulted in low variation in the Shannon index and log evenness, and due to the fact that only *Ae*. *aegypti* and *Cx*. *quinquefasciatus* were collected at this site there was no variation in the log abundance (Fig 6A). Even though more variation was present in the cumulative SHE analysis of the Golden Glades control site, the results indicated low levels of variability in the mosquito diversity.

The mosquito community composition was significantly different in the comparison between each urban farm and their respective control sites: Wynwood urban farm (Kruskal-Wallis, chi-squared = 13.9, df = 2, *P* < 0.001); Golden Glades urban farm (Kruskal-Wallis, chi-squared = 552.7, df = 11, *P* < 0.001).

## Discussion

The availability of environmental resources is vital to sustaining populations of vector mosquito species. The availability of these resources varies greatly in urban environments driving vector mosquito population dynamics, presence, and abundance [5,16,44,45].

Our results showed that mosquito species were abundantly found breeding and dwelling in urban farms, although they varied greatly in the community composition and abundance. The lower species richness found in the Wynwood urban farm, with *Cx*. *quinquefasciatus* comprising 90% of all specimens collected, may be explained due to its location in a densely populated and highly urbanized area [1,15,16,32,46]. *Ae*. *aegypti* and *Cx*. *quinquefasciatus* were collected at all stages in their aquatic stages, as resting adults, and females seeking a host for blood-feeding.

On the other hand, the Golden Glades urban farm displayed a higher species richness and a more even mosquito assembly with 5 species comprising 90% of all mosquitoes collected: *Ae*. *aegypti*, *Ae*. *albopictus*, *Cx*. *coronator*, *Cx*. *nigripalpus*, and *Cx*. *quinquefasciatus*. Among the total 12 species, *Ae*. *aegypti*, *Ae*. *albopictus*, *Cx*. *quinquefasciatus*, and *Wy*. *mitchelli* were collected as immatures, resting and flying adults, while *Ae*. *taeniorhynchus*, *Ae*. *tortillis*, *An*. *quadrimaculatus*, *Cx*. *coronator*, *Cx*. *erraticus*, *Cx*. *nigripalpus*, *Deinocerites cancer*, and *Wy*. *vanduzeei* were only collected by the BG-Sentinel trap. One possible explanation is that these species may have actively invaded the urban farm seeking for hosts.

The relatively low abundance of immature mosquitoes found in both the Wynwood and Golden Glades urban farms compared to the number of adult mosquitoes may imply that mosquitoes are actively moving into the urban farms seeking resources in its premises such as sugar and blood sources widely available in these environments. However, the presence of cryptic breeding sites cannot be excluded.

*Ae*. *albopictus* was the exception. Despite not being abundantly found in Miami, this species was able to use a wider range of natural aquatic habitats within the Golden Glades urban farm. *Ae*. *albopictus* was found breeding in high numbers in aquatic habitats at the Golden Glades urban farm in 5 of the 7 weeks sampled, but only a few adults were collected by the BG-Sentinel trap. The urban farms surveyed in this study were relatively small and BG-Sentinel traps have been specially designed to collect *Ae*. *aegypti* and *Ae*. *albopictus* mosquitoes [34]. Therefore, it was expected that the number of immature *Ae*. *albopictus* would be proportional to the number of adults, even more, due to the presence of pupae indicating their ability to reach adulthood. We hypothesize that immature *Ae*. *albopictus* mosquitoes are exploiting the resources available within the urban farm but once they reach adulthood they are seeking hosts elsewhere.

The comparison of the urban farms with their respective control sites revealed a distinct scenario. *Culex quinquefasciatus* was substantially more abundant in the Wynwood urban farm when compared to the control site. This result indicates the availability of suitable aquatic breeding habitats and the essential resources needed to sustain the development of *Cx*. *quinquefasciatus* population on the farm. As a consequence, the mosquito community composition is highly uneven, as highlighted by the Simpson index, being comprised mostly by *Cx*. *quinquefasciatus*.

The comparison between the Golden Glades urban farm to the control site revealed a much more diverse mosquito community composition. This finding indicates the availability of not only suitable aquatic breeding sites but also the availability of essential resources (e.g., sugar sources) making it possible for the establishment of many different mosquito species. This result was supported by the Shannon index indicating a more even and diverse mosquito community.

Favorable environments for mosquito proliferation such as sugar and blood sources and resting and aquatic habitats present in urban farms stand as a substantial challenge for public health and for the development of effective mosquito control strategies. Urban farmworkers spend the majority of their working days outdoors and are substantially exposed to vector mosquitoes and potentially to arboviruses [47]. The complex interaction between human behavior, weather conditions, and the inherent physical features present in urban farms may have a significant influence on the population dynamics of vector mosquitoes. Moreover, urban farms are often no-spray zones and chemical interventions to reduce mosquito populations are limited to emergency situations, increasing the complexity of the development of mosquito control strategies.

The diversity analyses showed that the sampling sufficiency was reached in both urban farms, even though the survey period was very short, suggesting no more species should be found in neither farm with an increase in the sampling effort. *An*. *quadrimaculatus* was collected only once with the aspirator, therefore, it could be considered an occasional capture. However, we were not able to collect data over long periods of time and across all weather and season variations that would further enhance insight into vector abundance and seasonality. We have in this paper included controls supporting the conclusion that urban farms are favorable habitats; in the previous papers [17,18,24,25] these kinds of controls were not included.

## Conclusion

Our results show how urban farms provide favorable environments for populations of vector mosquito species because they likely provide a wide range of essential resources needed for their survival. The increasing trend of urban agriculture and the increase in the numbers of urban farms represent a new challenge for the development of effective strategies to control populations of vector mosquito species in urban areas.
